#  Dosage Compensation of Sex Chromosome Genes in Eukaryotes 

**Published:** 2010

**Authors:** E.V. Dementyeva, S.M. Zakian

**Affiliations:** Institute of Cytology and Genetics, Siberian Branch, Russian Academy of Sciences; Institute of Chemical Biology and Fundamental Medicine, Siberian Branch, Russian Academy of Sciences; Research Center of Clinical and Experimental Medicine, Siberian Branch, Russian Academy of Medical Sciences

**Keywords:** dosage compensation, sex chromosomes, gene expression, X-chromosome inactivation

## Abstract

Sex chromosome evolution is accompanied by significant divergence in morphology and gene content and results in most genes of one of the sex chromosomes being present in two dosages in one sex and in one dosage in the other. To eliminate the difference in the expression levels of these genes between sexes and to restore equal expression levels of the genes between sex chromosomes and autosomes, mechanisms of dosage compensation have appeared. Studies of three classical objects,*Drosophila melanogaster*,*Caenorhabditis elegans*, and mammals, have shown that dosage compensation of X-linked genes can be achieved through completely different chromosome-wide mechanisms. New data on sex chromosome gene expression demonstrating that many sex chromosome genes can be expressed at different levels in males and females were recently obtained from birds and butterflies. In this review, dosage compensation mechanisms in*D. melanogaster*,*C. elegans*, and mammals are considered and the data on sex chromosome gene expression in birds and butterflies, and their influence on our view of dosage compensation, are discussed.

##  Coevolution of Sex Chromosomes and Dosage Compensation Mechanisms 

**Fig. 1:**
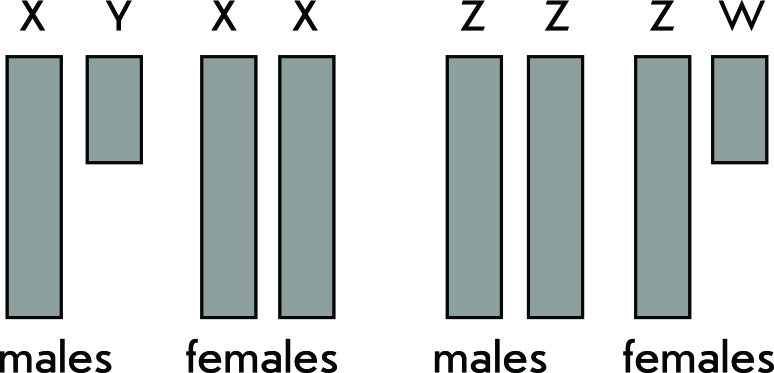
XY and ZW sex chromosome systems.

In a variety of organisms, sex correlates with a distinct sex chromosome set. In particular, in *Drosophila melanogaster,* as well as in most mammals, females have two X-chromosomes, while males are heterogametic with two different sex chromosomes, X and Y. Nonetheless, the systems determining sex in *D. melanogaster* and mammals are completely different. In *D. melanogaster* , sex depends on the ratio between doses of X-linked and autosomal genes [1], whereas in mammals the presence of the Y-chromosome, rather the *Sry* gene responsible for male sex determination, is crucial [2]. In contrast, in birds, butterflies, and some reptiles, females are heterogametic (chromosomes Z and W), while males have two Z-chromosomes. The sex chromosomes X and Y, as well as Z and W, considerably differ from each other in size, morphology, and gene content (Fig. 1). The chromosomes Y and W are heterochromatinized and mainly composed of tandem DNA repeats, and their gene content is poor in comparison with that of X- and Z-chromosomes.

It is thought that X- and Y-chromosomes appeared independently in different taxa and originated from a pair of homologous autosomes. The first step in sex chromosome evolution was the development of a genetic system of sex determination in a population of hermaphrodites or individuals whose sex is determined by temperature. The most consistent is an order of events with initial mutation leading to the appearance of a recessive gene of male sterility on the future X-chromosome, followed by the appearance of a dominant gene of female sterility on the future Y-chromosome. This resulted in the suppression of recombination between the X- and Y-chromosomes at the loci, which enabled the linkage of the genes responsible for male or female sex determination. The following step was the accumulation of genes beneficial to males (but decreasing the female’s fitness) on the Y-chromosome. The necessity of a tight linkage between these genes and the Y-chromosome resulted in the suppression of recombination between the X- and Y-chromosomes in new loci and gradual expansion of the nonrecombining region. Suppression of recombination led to the accumulation of mutations and deletions in Y-linked genes, which are not associated with the formation of male features, thereby resulting in their degradation. Finally, the entire Y-chromosome could be lost, which probably occurred in *Caenorhabditis elegans* males that only possess the X-chromosome. A similar process is likely to have resulted in the divergence of Z- and W-chromosomes [3, 4].

To compensate for such an essential loss of genes on the Y-chromosome, natural selection might have favored mechanisms that elevated the expression of X-linked genes in males [5]. Up-regulation of the genes localized on the single X-chromosome in males has been known for a long time and is well-studied in *D. melanogaster* [6]. A similar path of restoring the X-linked gene expression level (dosage compensation) was proposed for mammals and *C. elegans* , but convincing arguments took a long time to emerge. A hypothesis on X-chromosome up-regulation in mammal and *C. elegans* males was recently confirmed thanks to the development of microarray techniques. This method allowed to determine the mean expression level of autosomal and X-linked genes, which was found to be equal in males of both mammals and *C. elegans* [7–9].

**Fig. 2:**
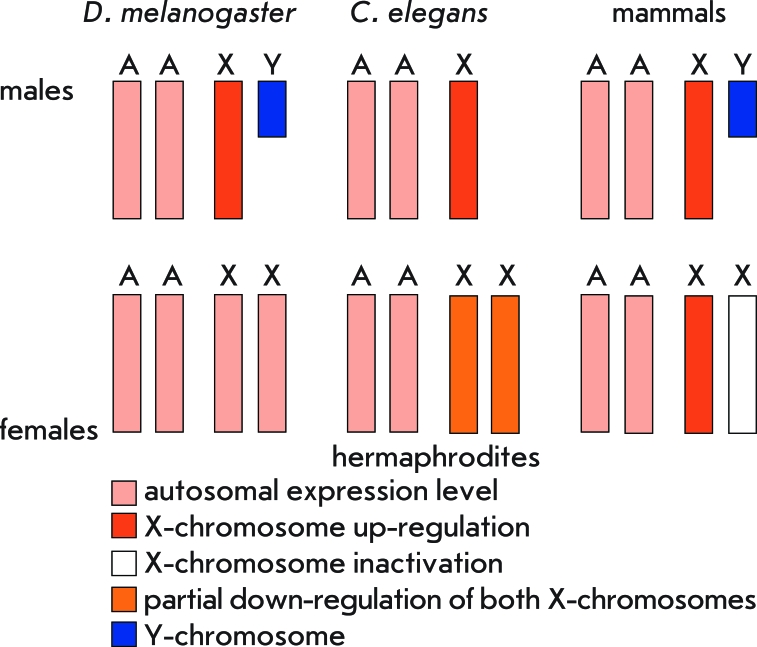
Diversity of X-linked gene dosage compensation systems A – autosomal set, X and Y – sex chromosomes.

An increase in the transcription level of X-linked genes might result in an excess of their products in females. However, studies on gene expression using microarray have shown that X-linked genes are expressed in females of *D. melanogaster* , *C. elegans* , and mammals at the same level as autosomal genes [7–9]. Hence, females should also possess the mechanism(s) supporting the transcription balance between the X-linked and autosomal genes, as well as an equal expression level of the X-linked genes in both sexes. Despite the fact that it has a similar origin, dosage compensation of X-linked genes occurs in different ways in *D. melanogaster* , *C. elegans* , and mammals (Fig. 2). In *D. melanogaster* , dosage compensation only occurs in males, while in females the expression levels of genes localized on the autosomes and both X-chromosomes are equal [7]. In *C. elegans* , the single X-chromosome in males and both X-chromosomes in hermaphrodites are up-regulated. The restoration of the transcription balance in hermaphrodites is achieved by a specific mechanism that partially down-regulates gene expression on both X-chromosomes [7]. In mammals, gene expression is up-regulated on the X-chromosome in males and one of the two X-chromosomes in females. On the second X-chromosome, transcription of most genes is completely repressed; i. e., this X-chromosome undergoes inactivation [7, 8]. The mechanisms underlying these processes deserve a more detailed examination.

## Dosage Compensation of X-linked Genes in * D. melanogaster*

The elevated level of expression of X-linked genes in *D. melanogaster* males is supported by a complex composed of six proteins: MSL1 (male-specific lethal 1), MSL2, MSL3, MOF (males absent on the first), MLE (maleless), and JIL1 (Janus kinase 1), and two noncoding RNAs: *roX1* and *roX2* (RNA on the X). The key element for the assembly of this complex is MSL2, which is only synthesized in males. The MSL2 protein is absent in females, so other components cannot form the dosage compensation complex. According to the generally accepted model, MSL2 stabilizes MSL1 by direct interaction, thus forming a platform for further assembly of the dosage compensation complex [10]. The proteins MOF and JIL1 are responsible for the activation of X-linked gene transcription in males. The MOF protein acetylates histone H4 at Lys16 (H4K16). This modification is characteristic of the transcriptionally active chromatin and specific for the male X-chromosome [11, 12]. However, recent data suggest that MOF can acetylate H4K16 not only on the X-chromosome, but also on the autosomes of both sexes [13]. MOF has been found to interact not only with the MSL complex, but also with the so-called NSL (nonspecific lethal) complex that binds to promotors of transcriptionally active autosomal genes in males as well as autosomal and sex-chromosomal genes in females. Hence, MOF, via interaction with different protein complexes, is implicated in two processes: dosage compensation of X-linked genes in males and general regulation of gene transcription in *D. melanogaster* [14, 15]. Moreover, homologues of MOF and the NSL complex exist in mammals, in which they play the same role in the histone H4 acetylation [16, 17]. These facts suggest that the mechanism of dosage compensation in *D. melanogaster* did not appear *de novo* but was formed on the basis of existing proteins which could retain their initial functions. JIL1 kinase is freely associated with the MSL complex and phosphorylates histone H3 at Ser10. This modification is also implicated in the formation of transcriptionally active chromatin, likely counteracting the binding of the heterochromatin protein HP1 [18, 19]. Thus, up-regulation of X-linked genes in *D. melanogaster* is achieved through the creation of an “open” incompact chromatin structure accessible to transcription factors [20]. RNA-DNA-helicase MLE is thought to promote the integration of *roX1* and *roX2* RNAs into the dosage compensation complex [10]. These RNAs are interchangeable and essential for the binding of the dosage compensation complex with the X-chromosome [21]. Interestingly, the human homologue of the MSL complex does not contain the *roX1* and *roX2* RNAs. So, the recruitment of these noncoding RNAs into the MSL complex might be the turning point in the formation of the dosage compensation mechanism in *D. melanogaster* [22].

The X-chromosome of *D. melanogaster* has no less than 150 specific sites, called chromatin entry sites, which contain MSL recognition elements for the binding of the dosage compensation complex. Following the binding of these sites, the MSL complex spreads along the X-chromosome and interacts with actively transcribed genes [23]. Epigenetic features, such as trimethylated H3K36 (a characteristic of transcribed genes), rather than nucleotide sequences, are most likely significant at this stage [24]. Nevertheless, not all transcriptionally active genes of the *D. melanogaster* male X-chromosome bind the dosage compensation complex. Moreover, binding of the MSL complex not always leads to exactly a twofold increase in the X-linked gene expression level. In some cases, the level of transcription remains virtually unchanged [25–27]. Therefore, the mechanism controlling the expression level of individual X-linked genes in *D. melanogaster* males has yet to be identified.

The mechanism underlying up-regulation of X-linked genes in mammalian and *C. elegans* males remains unknown. It is likely to be supported by epigenetic mechanisms as in *D. melanogaster* . However, it is worth noting that no significant difference has been found between the chromatin structures of the X-chromosome and autosomes. So, the X-chromosome up-regulation in males may be a result of alterations in the nucleotide sequences of the gene regulatory regions that took shape during evolution [8, 28]. Besides, there is another possible way of enhancing X-linked gene expression in mammals. The fact is that the genes of the active and inactive X-chromosomes differ in methylation patterns. The alleles of the inactive X-chromosome are hypermethylated at the CpG-dinucleotides of promotor regions, which matches their inactivation. At the same time, the alleles of the active X-chromosome in females and genes of the X-chromosome in males are hypermethylated at the CpG-dinucleotides of gene bodies [29]. However, it remains absolutely unclear how methylation of gene bodies can lead to elevated expression of X-linked genes in mammals.

## Dosage Compensation of X-linked Genes in * C. elegans*

As mentioned above, dosage compensation of the *C. elegans* X-linked genes includes two processes: X-linked gene up-regulation in males and partial repression of the genes localized on both X-chromosomes in hermaphrodites. While the mechanism of the first process is absolutely unknown, the complex of dosage compensation composed of nine proteins SDC-1, SDC-2, SDC-3, DPY-21, DPY-26, DPY-27, DPY-28, DPY-30, and MIX1 has been characterized in *C. elegans* hermaphrodites [30]. Three proteins (DPY-26, DPY-27, and DPY-28) closely resemble the proteins of the 13S condensin complex responsible for chromosome compaction in mitosis and meiosis not only in *C. elegans* , but also in other eukaryotes. Another protein, MIX1 (mitosis and X-associated protein 1), is common to both complexes [31–34]. However, not only MIX1 has a dual function. The protein DPY-28 controls the number and distribution of crossovers between homologous chromosomes in meiosis [35]. DPY-30 is part of a complex that is homologous to the yeast complex Set1/COMPASS methylating histone H3. DPY-30 is likely implicated in both dosage compensation and the general regulation of gene transcription in *C. elegans* males and hermaphrodites [36, 37]. An important role in the assembly and function of the dosage compensation complex is played by the protein SDC-2 (sex determination and dosage compensation 2). Unlike other proteins, SDC-2 is only expressed in hermaphrodites and seems to be responsible for the specific impact of the dosage compensation complex on the X-chromosome, because it can bind to the X-chromosome independently of other components of the complex [38]. The complex assembly begins with the interaction between SDC-2, SDC-3, and DPY-30, which creates a platform for the binding of all other proteins to the X-chromosome [39–41]. Interestingly, the same complex (except for DPY-21) is implicated in a 20-fold transcription repression of the autosomal gene *her-1* (hermaphrodization of X0 animals), which is responsible for male sex determination [38, 41]; i.e. this complex participates not only in the dosage compensation of X-linked genes, but also in the sex determination system.

**Fig. 3:**
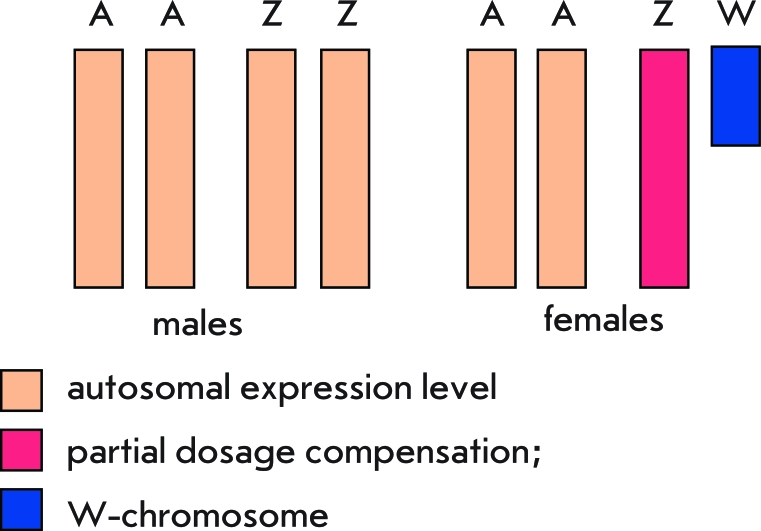
Dosage compensation of Z-chromosome genes in birds and butterflies A – autosomal set, Z and W – sex chromosomes.

To bind to the dosage compensation complex there are specific nucleotide sequences on the *C. elegans* X-chromosome, but their density is significantly lower than that on the *D. melanogaster* X-chromosome (~40 and 150, respectively). These sequences are divided into two types: the rex- and dox-sites. Rex (recruitment elements on X)-sites can bind to the dosage compensation complex regardless of whether they are localized on the X-chromosome or autosomes and are most likely responsible for the primary recognition of the complex. Dox (dependent on X)-sites only interact with the dosage compensation complex when localized on the X-chromosome, and they are mainly implicated in the spreading of the complex along the X-chromosomes of *C. elegans* hermaphrodites [42].

The mechanism of dosage compensation complex-dependent partial repression of X-linked gene expression in hermaphrodites is not yet known; however, a certain similitude between the *C. elegans* dosage compensation and the 13S condensin complexes allows to assume that the same principle underlies both the transcription repression of X-linked genes and the chromosome condensation in mitosis and meiosis [37]. The similarity to the 13S condensin complex and the dual function of some proteins of the dosage compensation complex suggest that in both *C. elegans* and *D. melanogaster* dosage compensation appeared due to the acquisition of novel functions by existing proteins, rather than the development of an absolutely new mechanism. Which X-linked genes are subjects to dosage compensation in *C. elegans* hermaphrodites (and to what extent) remains unknown.

##  Dosage Compensation of X-linked Genes in Mammals 

Like *C. elegans* , mammals demonstrate up-regulation of X-linked genes in both sexes. The restoration of the gene transcription balance in females is achieved by transcription repression (inactivation) of the majority of genes localized on one of the two X-chromosomes [43]. X-inactivation may be either random or imprinted [28]. When X-inactivation is imprinted, the paternally inherited X-chromosome is predominantly inactivated. This variant of inactivation occurs in marsupials as well as in the extraembryonic tissues of some eutherians. When the inactivation is random, the chances of the paternal and maternal X-chromosomes being inactivated are equal. This type of X-inactivation takes place in somatic tissues of eutherians.

The eutherian X-chromosome has a specific locus called the X-inactivation center. One gene from this locus, *Xist* , is the key gene in the initiation of the X-inactivation process. It encodes a noncoding RNA, which then spreads along the further inactive X-chromosome, which leads to a series of epigenetic changes [28, 44–46]. As a result, RNA polymerase II is excluded from the inactive X-chromosome, and chromatin-modifying complexes appear. Consequently, the inactive X-chromosome loses modifications that are characteristic of transcriptionally active chromatin, such as histone H3 dimethylated at Lys4 (Н3К4) and acetylated histones H3 and H4. Instead, the inactive X-chromosome gains modifications characteristic of transcriptionally inactive chromatin, such as histone H3 trimethylated at Lys27 (Н3К27), histone H2A ubiquitinylated at Lys119 (uH2A), histone H3 dimethylated at Lys9 (Н3К9), and histone H4 monomethylated at Lys20 (Н4К20). In addition, the inactive X-chromosome becomes late-replicating and associates with a histone H2A variant (macroH2A) containing a nonhistone domain. The last epigenetic event in the X-inactivation process is methylation at X-linked gene promotor regions, which allows to maintain the stability of the inactive state of the X-chromosome. Complexes of polycomb proteins are implicated in the establishment of the inactive state of the X-chromosome in mammalian females. PRC1 (polycomb repressor complex 1) is responsible for the ubiquitinylation of histone H2A [47, 48], while PRC2 is responsible for Н3К27 trimethylation [49, 50]. However, these complexes are not specific to females, as they are also implicated in the repression of both X-linked and autosomal genes [22]. The enzymes fulfilling Н3К9 dimethylation and Н4К20 monomethylation have not been known precisely. It is believed that they are methyltransferase G9a and PR-Set7, respectively [51, 52]. It is worth noting that noncoding RNAs and chromatin-modifying complexes are involved in the dosage compensation of X-linked genes both in mammals and *D. melanogaster* , but their effects on gene expression are diametrically opposite. The question of how *Xist* RNA interacts with chromatin-modifying factors still remains open. Moreover, the gene *Xist* was not found in marsupials [53], despite the fact that their chromatin modification patterns on the inactive X-chromosome closely resemble those in eutherians. It becomes obvious that both the X-inactivation center and random X-inactivation only developed in eutherians [53, 54], and that the X-inactivation process in marsupials differs from that in placental mammals.

In its mechanism, X-inactivation is similar to the imprinting of autosomal genes. In both cases, noncoding RNAs are involved whose expression leads to the establishment of the same chromatin modifications: hypomethylated Н3К4, hypoacetylated Н3К9, trimethylated Н3К27, uH2A, dimethylated Н3К9, and DNA methylation [55, 56]. The final result of both processes is the transcription repression of one of two alleles. Hence, the method of gene transcription repression during X-inactivation in mammalian females is not unique; the same mechanism is also at play in the establishment of monoallele expression for certain autosomal genes.

It is worth noting that not all genes of the inactive X-chromosome undergo inactivation. Studies on the expression status of human X-linked genes have shown that 15% of genes always escape X-inactivation, while 10% of genes have heterogeneous expression; i.e., they undergo X-inactivation in some women and escape X-inactivation in others [57]. Besides, genes escaping X-inactivation were found in mice and some other mammals [58, 59]. However, the reason why some X-linked genes escape X-inactivation is as yet unknown. In some cases, this may be explained by the presence of a Y-chromosomal homologue of an X-linked gene. In this case, escaping X-inactivation enables the restoration of equal X-linked gene expression between the sexes. Nonetheless, many X-linked genes escaping X-inactivation have no Y-homologues. It is possible that a higher expression level of these genes in females is associated with the formation of female-specific features [60, 61]. Interestingly, the expression level of many genes escaping X-inactivation on the inactive X-chromosome is much lower than that on the active X-chromosome [8, 9, 57]. This suggests that a higher expression level of these genes in females is of little importance. It is also possible that an imbalance of X-linked genes can be evened out after transcription [60, 61].

It has been suggested that special elements are necessary for the effective spreading of the inactive state along the X-chromosome. The most likely candidates are long, interspersed nuclear elements (LINEs) [62]. This hypothesis is supported by the fact that murine and human X-chromosomes are twice richer in LINEs as compared to autosomes. It is worth noting that the distribution of LINEs on the human X-chromosome correlates with gene expression status. The highest density of LINEs is observed in the X-inactivation center and in regions of gene inactivation. Conversely, the density of LINEs in regions that escape X-inactivation is lower [57, 63, 64]. Yet, what are the sequences necessary for effective spreading of the inactive state along the X-chromosome and what are the mechanisms of their action remains unknown.

##  Common Features of X-linked Gene Dosage Compensation Systems 

Examination of three model objects ( *D. melanogaster* , *C. elegans* , and mammals) demonstrates that X-linked gene dosage compensation can occur via a variety of mechanisms. The difference in dosage compensation mechanisms appears to reflect an independent origin of sex chromosomes in these species and, as a result, independent formation of the mechanisms directed toward the regulation of X-linked gene expression. Despite the difference in means of X-linked gene dosage compensation in *D. melanogaster* , *C. elegans* , and mammals, there are several common features. First, dosage compensation is achieved via mechanisms operating at the chromosomal level; these mechanisms do not appear *de novo* : existing proteins and protein complexes adapt to the regulation of X-linked gene expression. Second, up-regulation of the single X-chromosome in males is common to all three dosage compensation systems, though the mechanisms underlying this phenomenon may differ. Third, the required gene expression level is supported via a change in the X-chromosomal chromatin structure by chromatin-modifying complexes. In *D. melanogaster* and mammals, the effect of chromatin-modifying complexes in the course of X-linked gene dosage compensation is associated with the expression of noncoding RNAs. Tight association between the noncoding RNAs and regulation of gene expression implies that noncoding RNA is likely to be also found in the *C. elegans* dosage compensation system. Fourth, the X-chromosome contains a set of sequences responsible for binding and effective spreading of the dosage compensation complexes. Thus, the mechanisms of dosage compensation enable the leveling of the expression of autosomal and X-linked genes, as well as maintenance of an equal expression level of X-linked genes in both sexes. Transcriptional balance of X-linked genes is supported in different somatic cell types and the germinal cells of *D. melanogaster* and mammals in both sexes [7, 8], thus suggesting its importance to the organism.

##  Dosage Compensation of Z-linked Genes in Birds and Butterflies 

By analogy with the XY system of sex chromosomes, one might expect that the ZW system of sex chromosomes should also be characterized by the up-regulation of genes on the single Z-chromosome in females (heterogametic sex). However, early studies on the expression of a small number of Z-linked genes in birds and butterflies showed an elevated expression of some Z-linked genes in males, as compared to females [65–68]. Thus, the existence of Z-linked gene dosage compensation was called into doubt for a long time.

The use of microarray techniques allowed to determine the level of Z-linked and autosomal gene expression in two avian species (chicken and zebra-finch) and in silkworm. The ratio between male and female Z-linked gene expression levels in birds ranged between 1 and 2: i.e., one gets the impression that the Z-chromosome is between dosage compensation at the chromosomal level and the lack of dosage compensation [69, 70]. Similar data was obtained from the study of Z-linked gene expression in silkworm [71]. Moreover, in zebra-finch, Z-linked genes were distinctly divided into two groups: genes with an equal expression level in both sexes and those with a higher expression level in males [69]. Birds and butterflies do not appear to have mechanisms controlling the gene expression of the whole Z-chromosome; however, some Z-linked genes in females do undergo dosage compensation (Fig. 3). The mechanisms involved in this process are not yet understood. Nonetheless, a specific locus, MHM (male hypermethylated), has been found on the avian Z-chromosome. This locus is hypermethylated in males, while in females a noncoding RNA is transcribed from this locus and accumulates in the region surrounding MHM. In females, this region is acetylated at Lys16 of histone H4 (H4K16). Besides, despite the genes undergoing dosage compensation being distributed along the Z-chromosome, the majority of them are concentrated near the MHM locus. It is likely that Z-linked gene dosage compensation in birds occurs the same way it does in *D. melanogaster* : noncoding RNA and Н4К16 acetylation provide an elevated expression level of Z-linked genes in females [72].

The difference in the degrees of dosage compensation between the X- and Z-chromosomes might be ascribed to their age. When sex chromosomes are young enough, the mechanisms controlling gene expression might not have developed yet. While avian and mammalian sex chromosomes are close in age (no less than 150 and 166 MYA, respectively), the sex chromosomes of *D. melanogaster* are relatively young (~65 MYA), but the age is enough in order for the chromosomal dosage compensation mechanism to have developed. Hence, the age of sex chromosomes does not influence the extent of dosage compensation [22, 73]. It is known that hemizygosity at several genes or small genomic regions may remain, with no consequences for the organism. The avian and butterfly Z-chromosomes contain about 840 and 600 genes, respectively, which is considerably less than the number of genes on the *D. melanogaster* , *C. elegans* , and human X-chromosomes (2,300; 3,100; and 1,100 genes, respectively). It is likely that it is the lower number of genes on the sex chromosomes of birds and butterflies that allows them to do without chromosomal dosage compensation mechanisms. However, hemizygosity at several hundred genes must be lethal anyway; so the limited dosage compensation in birds and butterflies cannot be the result of lower gene density on sex chromosomes [22, 73]. As of now, the local dosage compensation is only found in species whose heterogametic sex is female (ZW). Since the Z-linked gene expression level was only examined in representatives of two taxa, it remains unclear whether this path of dosage compensation is characteristic of organisms with the ZW sex chromosome system or whether it is a coincidence. The study of other taxa, whose heterogametic sex is female, will likely answer this question [22, 73].

##  Conclusion 

The data on sex-chromosomal gene expression in birds and butterflies force us to look anew at the problem of gene dosage compensation. It is becoming obvious that sex-chromosomal genes undergo dosage compensation to different extents, up to its complete escaping. It is possible that dosage compensation mechanisms evolved to control the expression of a distinct gene set, rather than the entire sex chromosome. This postulate seems to be correct not only for the Z-chromosome, but also for the X-chromosome, because genes escaping dosage compensation were found in mammals and *D. melanogaster* . Further studies will probably focus on the identification of the sex-chromosomal genes requiring dosage compensation, as well as on the mechanisms that determine the extent of dosage compensation for individual genes. Another important line of inquiry may be uncovering the mechanisms underlying the up-regulation of the X-linked genes in mammals and *C. elegans* . Studies on heteromorphic sex chromosomes in new taxa could shed light on the matter.
